# Diagnostic value of derived neutrophil‐to‐lymphocyte ratio in patients with ovarian cancer

**DOI:** 10.1002/jcla.22833

**Published:** 2019-01-21

**Authors:** Yang‐yang Wu, Yuan‐yuan Qin, Jin‐qiu Qin, Xuan Zhang, Fa‐quan Lin

**Affiliations:** ^1^ Department of Clinical Laboratory The First Affiliated Hospital of Guangxi Medical University Nanning China

**Keywords:** benign ovarian disease, derived neutrophil‐to‐lymphocyte ratio, diagnosis, inflammatory biomarker, ovarian cancer

## Abstract

**Background:**

Inflammation plays an important role in the occurrence and development of cancer. Numerous studies have used the derived neutrophil‐to‐lymphocyte ratio (dNLR) to evaluate prognosis in many types of cancer. However, the relationship between dNLR and ovarian cancer and its value in the differential diagnosis of benign and malignant ovarian tumors remain unknown.

**Methods:**

A total of 262 patients with ovarian cancer, 258 with benign ovarian disease, and 232 healthy controls were included in this study. dNLR was calculated using whole blood cell parameters. Receiver operating characteristic curves were generated to obtain sensitivity, specificity, and area under the ROC curve (AUC) to evaluate the diagnostic values of dNLR.

**Results:**

dNLR was significantly different among the ovarian cancer, benign ovarian disease, and healthy control groups (all *P* < 0.001). Moreover, there were significant differences in dNLR between patients with early‐stage (I and II) and advanced‐stage (III and IV) disease (*P* < 0.001). dNLR was positively correlated with stage and carbohydrate antigen‐125 in ovarian cancer. A cutoff value of dNLR ≤2.11 was diagnostic in distinguishing ovarian cancer from benign ovarian disease with AUC of 0.729 (95% confidence interval [CI], 0.689‐0.767; *P* = 0.0001). A cutoff value of dNLR ≤1.9 was diagnostic in distinguishing ovarian cancer from healthy controls with an AUC of 0.821 (95% CI, 0.784‐0.854; *P* = 0.0001).

**Conclusion:**

dNLR may be a useful indicator for distinguishing between ovarian cancer and benign ovarian disease and for identifying early and advanced ovarian cancer.

## 1 INTRODUCTION

Ovarian cancer is one of the most common malignant tumors of the female reproductive organs, with an incidence that ranks third after cervical cancer and uterine body cancer.^1^, ^2^ However, the mortality rate of ovarian cancer is the highest among malignant gynecological tumors, which seriously endangers women's health.^3^ Because the ovaries are located deep in the pelvic cavity and are not easily palpated, and early‐stage ovarian cancer often has no obvious symptoms, 70% of patients with ovarian cancer have advanced‐stage disease at the time of diagnosis.^4^, ^5^ In addition, although therapies such as surgery and chemotherapy continue to improve, the 5‐year survival rate of patients with advanced ovarian cancer remains approximately 30%, and the prognosis is poor.^5^, ^6^

Ovarian cancer is the fifth leading cause of death in American women. The number of new ovarian cancer cases and deaths in the United States in 2017 was estimated at 22 400 and 14 080, respectively.^7^ The incidence of gynecologic cancer is rising in India, and an estimated 36 199 women will have ovarian cancer in 2020, accounting for 19.8% of all gynecologic cancers.^8^ The population in China is gradually aging, and the incidence of ovarian cancer is rising. In China, the estimated number of new ovarian cancer cases and deaths in 2013 was 50 000 and 21 300, respectively and in 2015 was 52 100 and 22 500, respectively.^9^, ^10^

Inflammation plays an important role in the occurrence and development of cancer.^11^, ^12^, ^13^ Therefore, cancer is also widely considered a cause chronic inflammation. Many biomarkers can detect a systemic inflammatory state, including dNLR, C‐reactive protein, C‐reactive protein‐albumin ratio, and cytokines such as IL‐6 and TNFα.^14^, ^15^, ^16^ However, C‐reactive protein and cytokines are not routinely tested, so they are not widely used in clinical practice.^17^ The dNLR is calculated by whole blood cell parameters. Most patients in hospital or outpatient departments undergo routine blood tests, so it is easy to obtain whole blood cell parameters in routine laboratory testing, and the cost is relatively inexpensive.^17^

dNLR has been used to evaluate prognosis in many types of cancer, including gastric cancer, pancreatic cancer, colon cancer, and breast cancer.^13^, ^18^, ^19^, ^20^ However, the potential diagnostic value of dNLR in ovarian cancer remains unclear. The purpose of this study was to investigate the relationship between dNLR and ovarian cancer and to explore its value in the differential diagnosis of benign and malignant ovarian tumors.

## 2 MATERIALS AND METHODS

### 2.1 Study population

We retrospectively analyzed 262 patients with ovarian cancer who were diagnosed at the First Affiliated Hospital of Guangxi Medical University, China from August 2012 to July 2017. The inclusion criteria were as follows: (a) age ≥18 years and (b) complete surgical resection with a histologically confirmed diagnosis of ovarian cancer. The exclusion criteria were as follows: (a) cardiovascular disease, diabetes, acute inflammation, blood disease, kidney disease, or other cancers and (b) recent blood transfusion within the previous 3 months. Ovarian cancer stage was classified according to the International Federation of Gynecology and Obstetrics (FIGO) 2000. Patients diagnosed with benign ovarian disease in our hospital during the same period were included in the benign ovarian disease group. Healthy women who had undergone physical examination at the hospital were selected as the healthy control group. This study protocol was approved by the Ethics Committee of the Affiliated First Hospital of Guangxi Medical University, China. All the participants gave written informed consent.

### 2.2 Laboratory testing

Venous blood (2 mL) was collected on an empty stomach from each participant and placed into an EDTA‐K2 anticoagulant tube and drying tube. All whole blood cell parameters were measured by a Beckmann 780 hematology analyzer (Beckman Coulter, Brea, CA). The CA‐125 level was measured by the Roche E6000 analyzer (Roche Diagnostics, Basel, Switzerland). White blood cell count (WBC), hemoglobin, neutrophil count, lymphocyte count, and monocyte count were obtained directly from the hematology analyzer. The dNLR was calculated using the following ratio: neutrophil count:(WBC‐neutrophil count).^21^

### 2.3 Statistical analyses

Statistical analyses were performed using SPSS 20.0 (IBM Corp, Armonk, NY, USA) and GraphPad Prism 5 (GraphPad Software, San Diego, CA, USA) statistical software. Continuous variable data are expressed as means ± standard deviation or medians (quartile); categorical variable data are expressed as frequencies or rates. Data comparisons between two groups were performed using Student's *t* test or the Mann‐Whitney *U* test. Data comparisons among three groups were performed using one‐way analysis or the Kruskal‐Wallis *H* test. The chi‐square test was used to compare rates or frequencies. In the ovarian cancer group, the correlation between dNLR and stage or CA‐125 was analyzed with Spearman's test. A *P* value <0.05 was considered statistically significant. MedCalc version 15.0 (MedCalc Software, Mariakerke, Belgium) was used to draw receiver operating characteristic (ROC) curves. This software can calculate sensitivity, specificity, positive likelihood ratio, negative likelihood ratio, positive predictive value, negative predictive value, and area under the ROC curve (AUC), which were used to evaluate the diagnostic values of dNLR.

## 3 RESULTS

### 3.1 Patients and laboratory characteristics

A total of 262 patients with ovarian cancer, 258 with benign ovarian disease, and 232 healthy controls were included in the final analysis. Detailed information concerning the age and laboratory parameters of the study subjects is presented in Table 1. The age range of patients with ovarian cancer was 18‐81 years, of patients with benign ovarian disease ranged was 23‐71 years, and of healthy controls was 19‐73 years. The three groups did not significantly differ in age.

**Table 1:** Comparisons of laboratory parameters among the ovarian cancer, benign ovarian disease, and healthy controls

Parameter	Ovarian cancer	Benign ovarian disease	Healthy control	*P* value
n	262	258	232	
Age, y	43.48 ± 11.45	43.19 ± 9.46	42.82 ± 12.32	0.804
WBC, ×10^9^/L	7.99 ± 3.48*	6.85 ± 2.29**	6.09 ± 1.09***	<0.001
Hb, g/L	105.58 ± 20.46*	124.50 ± 13.04**	131.98 ± 7.29***	<0.001
Neutrophils, ×10^9^/L	5.76 ± 3.39*	4.21 ± 2.05**	3.43 ± 0.83***	<0.001
Lymphocytes, ×10^9^/L	1.54 ± 0.66*	2.03 ± 0.71**	2.09 ± 0.45	<0.001
Monocytes, ×10^9^/L	0.51 ± 0.20*	0.46 ± 0.16**	0.40 ± 0.10***	<0.001
CA‐125, U/mL	92.08 (34.15‐473.45)*	23.29 (13.10‐37.98)**	6.34 (4.06‐11.10)***	<0.001
dNLR	2.29 (1.50‐3.55)*	1.44 (1.14‐1.88)**	1.29 (1.06‐1.54)***	<0.001

Data are expressed as mean ± standard deviation or median (interquartile range).

CA‐125, carbohydrate antigen‐125; dNLR, derived neutrophil‐to‐lymphocyte ratio; Hb, hemoglobin; WBC, white blood cell count.

* *P* < 0.05, ovarian cancer group vs benign ovarian disease group.

** *P* < 0.05, ovarian cancer group vs healthy control group.

*** *P* < 0.05, benign ovarian disease group vs healthy control group.

### 3.2 Ovarian cancer stage and dNLR

**Figure 1:**
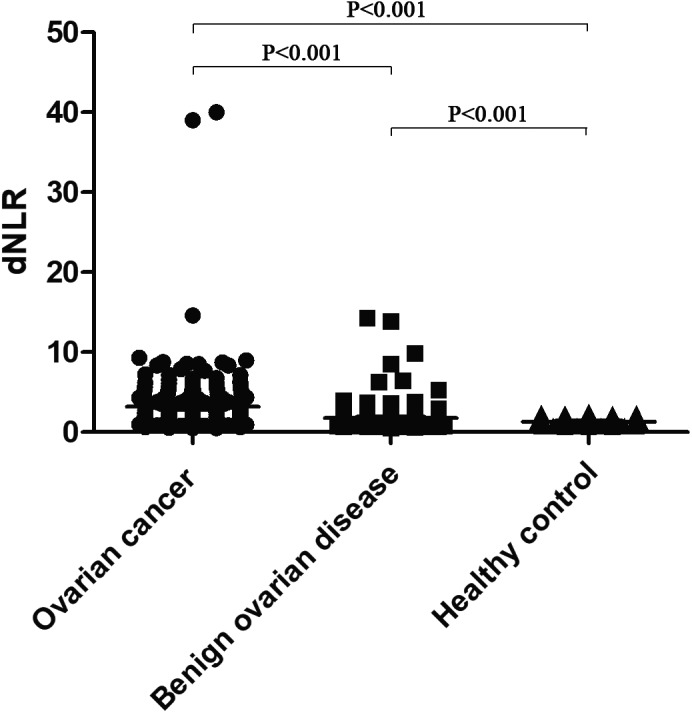
Derived neutrophil‐to‐lymphocyte ratio among women with ovarian cancer, benign ovarian disease, and healthy controls. dNLR, derived neutrophil‐to‐lymphocyte ratio

Detailed information concerning the ovarian cancer stage is presented in Table 2. The dNLR was significantly different between patients with early‐stage (I and II) and advanced‐stage (III and IV) cancer. Furthermore, the dNLR value was significantly different among the ovarian cancer, benign ovarian disease, and healthy control groups (all *P* < 0.001; Figure 1).

**Table 2:** Comparison of derived neutrophil‐to‐lymphocyte ratio (dNLR) according to ovarian cancer stage

Parameter	Stage I + II	Stage III +IV	*P* value
n	136	126	
dNLR	1.78 (1.21‐2.61)	3.07 (2.14‐4.79)	<0.001

### 3.3 Correlation between dNLR markers and stage and CA‐125 in ovarian cancer

Statistical correlation analysis showed a positive correlation between dNLR and stage in ovarian cancer (correlation coefficient: 0.507, *P* < 0.001). Furthermore, a positive correlation was found between dNLR and CA‐125 (correlation coefficient: 0.479, *P* < 0.001).

### 3.4 Diagnostic value of dNLR for distinguishing ovarian cancer from benign ovarian disease and healthy controls

**Figure 2:**
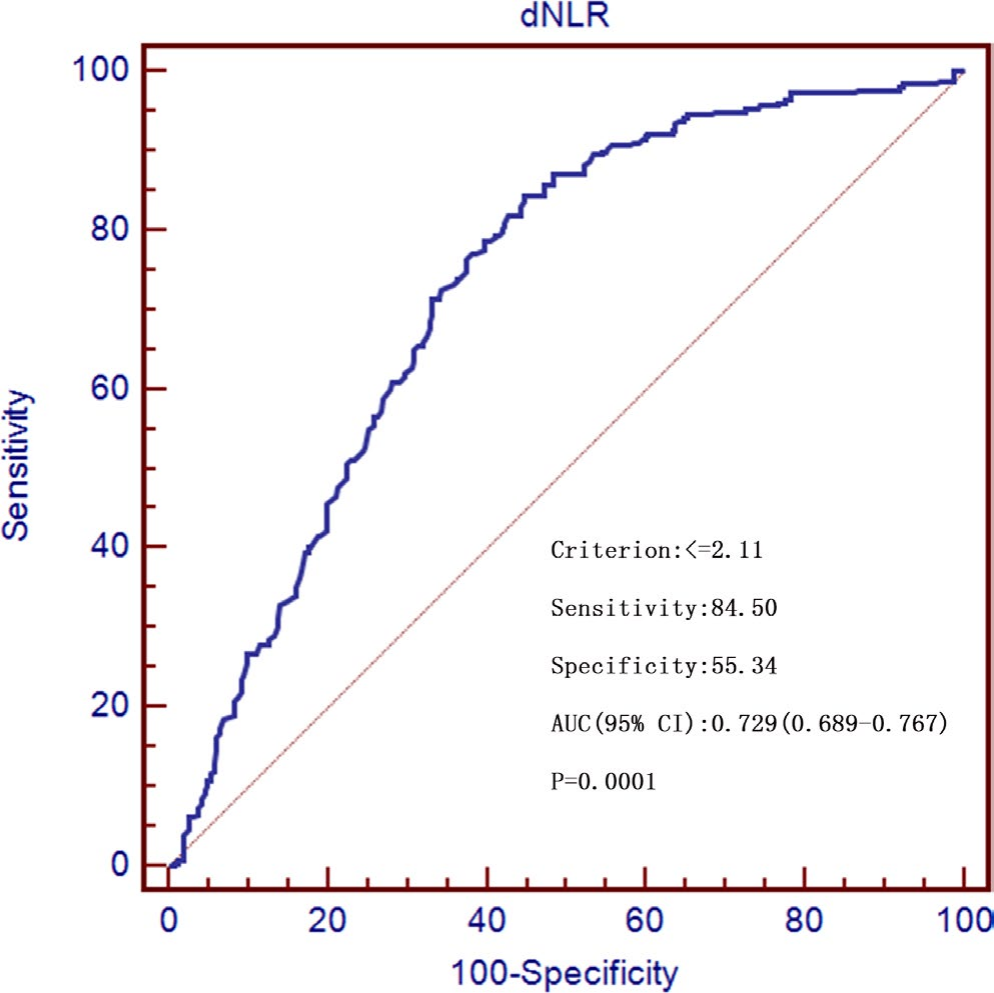
Receiver operating characteristics curves for preoperative derived neutrophil‐to‐lymphocyte ratio showing sensitivity and 100‐specificity for the differential diagnosis of ovarian cancer versus benign ovarian disease. AUC, area under the curve; dNLR, derived neutrophil‐to‐lymphocyte ratio

**Figure 3:**
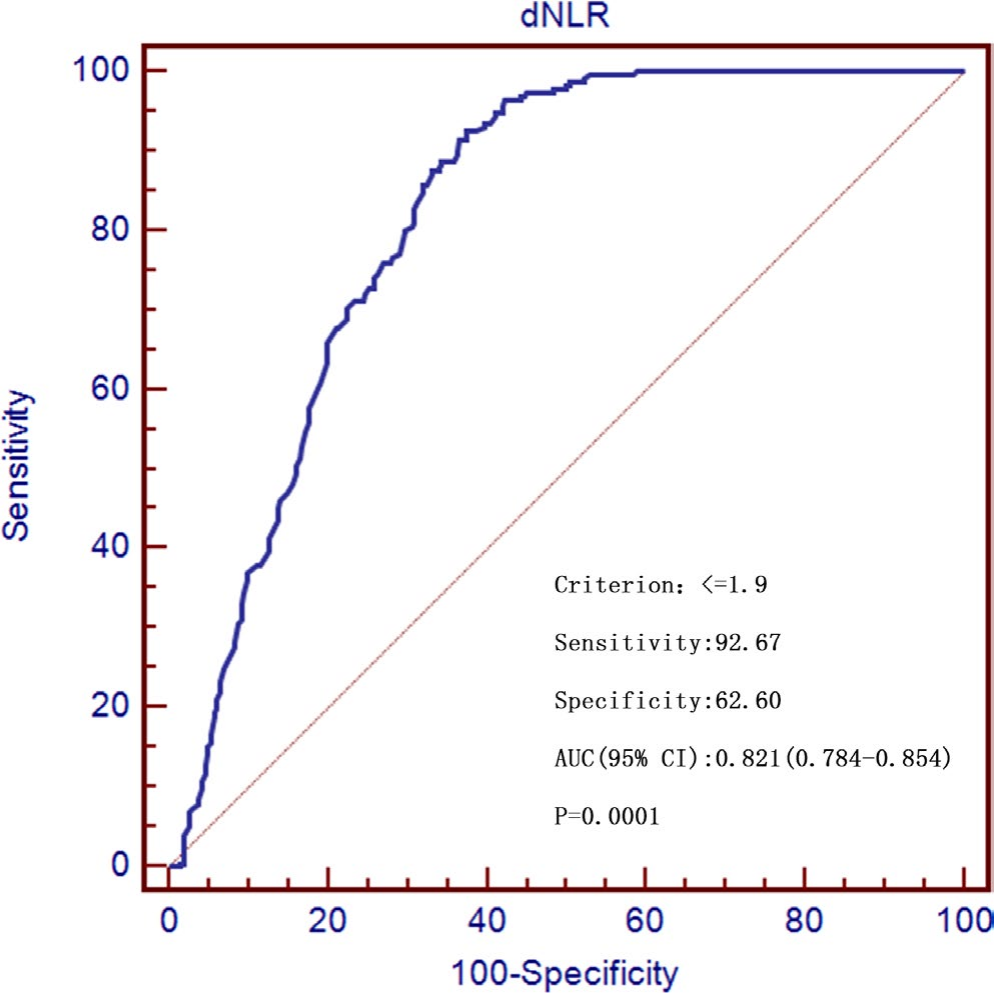
Receiver operating characteristics curves for preoperative derived neutrophil‐to‐lymphocyte ratio showing sensitivity and 100‐specificity for the differential diagnosis of ovarian cancer versus healthy controls. AUC, area under the curve; dNLR, derived neutrophil‐to‐lymphocyte ratio

As shown in Figures 2 and 3, at a cutoff of ≤2.11, dNLR was able to distinguish ovarian cancer from benign ovarian disease with a positive likelihood ratio, negative likelihood ratio, positive predictive value, and negative predictive of 1.89, 0.28, 65.1, and 78.4, respectively. At a cutoff of ≤1.9, dNLR was diagnostic for distinguishing ovarian cancer from healthy controls with a positive likelihood ratio, negative likelihood ratio, positive predictive value, and negative predictive value of 2.48, 0.12, 68.7, and 90.6, respectively.

## 4 DISCUSSION

The inflammatory response is one of the signature features of tumor development and plays an important role in tumor progression.^11^, ^22^ Research on the diagnosis and prognosis of various inflammatory cells has become a targeted area of interest in recent years. Studies have shown that inflammation can induce DNA, inhibit cell apoptosis, and promote angiogenesis in the surrounding area by releasing leukocytes and other phagocytic cell mediators or inflammatory cytokines, thus promoting the growth and development of tumors.^22^, ^23^ dNLR is one of the indicators of inflammation that has a prognostic value and relationship with clinicopathological features in patients with cancer.^13^, ^21^ In our study, the dNLR value in the ovarian cancer group was significantly higher than in the benign ovarian disease or healthy control groups. We also found that dNLR was significantly higher in the advanced‐stage (stage III and IV) ovarian cancer group compared to the early‐stage (stage I and II) ovarian cancer group. Therefore, we believe that dNLR may be an indicator in the differential diagnosis of benign and malignant ovarian tumors and may be used as a marker for early versus advanced ovarian cancer.

The specific mechanism by which dNLR affects the occurrence and development of malignant tumors is not yet clear, but the following to hypotheses have been proposed: (a) angiogenic factors play an important role in the progression and metastasis of cancer such as ovarian cancer, in which the increase in VEGF is associated with a decline in the survival rate of ovarian cancer.^11^, ^24^ Furthermore, studies have shown that neutrophils promote high energy secretion of oncostatin M and other factors to promote tumor invasion and metastasis and also release a large amount of active oxygen, leading to cancer.^25^, ^26^ (b) In the tumor microenvironment, tumor‐infiltrating neutrophils can differentiate into different phenotypes after tumor stimulation and promote or inhibit the development of tumors; cytokines and chemokines recruit neutrophils to the tumor microenvironment, where the neutrophils promote the release of several tumor growth factors. The antitumor activity of these factors is reduced to promote the development of tumor.^27^, ^28^

CA‐125 is a high molecular weight transmembrane glycoprotein similar to mucin.^29^ CA‐125 is a marker of ovarian cancer and has been widely used in the clinical diagnosis and postoperative monitoring of ovarian cancer.^3^, ^29^ This study found that there were significant differences in CA‐125 among the ovarian cancer group, the benign ovarian disease group, and the healthy control group, and CA‐125 was significantly increased in the ovarian cancer group, which is consistent with previous reports.^30^ We also found that there was a positive correlation between dNLR and CA‐125, and there was a correlation between dNLR and ovarian cancer staging. Therefore, we hypothesize that dNLR may also serve as a marker in the clinical diagnosis of ovarian cancer.

It is known that a greater AUC indicates better diagnostic accuracy. Moreover, AUC has a moderate accuracy at 0.7 to 0.9. In our study, a dNLR cutoff value of ≤2.11 distinguished ovarian cancer from benign ovarian disease, and a dNLR cutoff of ≤1.9 distinguished ovarian cancer from healthy controls with high sensitivity and moderate accuracy. Accordingly, we believe that the dNLR is a promising diagnostic biomarker for ovarian cancer.

There were several limitations in our study. First, this was a retrospective study of patients with ovarian cancer and benign ovarian disease, and some residual confounding factors could not be ruled out, possibly leading to a certain degree of deviation. Second, the study population came from a single center, and the results might not be representative of the rest of the population. Finally, there were fewer samples and studies of dNLR in the ovarian cancer group. Multicenter and longitudinal studies with larger sample sizes are needed to verify the association of dNLR with ovarian cancer. However, this study is the first to explore the relationship between dNLR and ovarian cancer, and its diagnostic value in ovarian cancer. Moreover, the study provides a reference for early detection and diagnosis of patients with ovarian cancer.

## AUTHORS' CONTRIBUTIONS

Yang‐yang Wu designed the study and wrote the initial draft of the paper. Yuan‐yuan Qin, Jin‐qiu Qin, and Xuan Zhang collected the data and carried out statistical analysis. Fa‐quan Lin revised the article.
